# Study on the Mechanism Underlying the Regulation of the NMDA Receptor Pathway in Spinal Dorsal Horns of Visceral Hypersensitivity Rats by Moxibustion

**DOI:** 10.1155/2016/3174608

**Published:** 2016-04-20

**Authors:** L. D. Wang, J. M. Zhao, R. J. Huang, L. Y. Tan, Z. H. Hu, Z. J. Weng, K. Wang, H. G. Wu, H. R. Liu

**Affiliations:** ^1^Key Laboratory of Acupuncture and Immunological Effects, Shanghai Research Institute of Acupuncture and Meridian, 650 South Wanping Road, Shanghai 200030, China; ^2^Shanghai TCM-Integrated Hospital, Shanghai University of TCM, Shanghai 200082, China

## Abstract

Visceral hypersensitivity is enhanced in irritable bowel syndrome (IBS) patients. Treatment of IBS visceral pain by moxibustion methods has a long history and rich clinical experience. In the clinic, moxibustion on the Tianshu (ST25) and Shangjuxu (ST37) acupoints can effectively treat bowel disease with visceral pain and diarrhea symptoms. To investigate the regulatory function of moxibustion on the Tianshu (ST25) and Shangjuxu (ST37) acupoints on spinal cord NR1, NR2B, and PKC*ε* protein and mRNA expression in irritable bowel syndrome (IBS) visceral hypersensitivity rats, we did some research. In the study, we found that moxibustion effectively relieved the IBS visceral hyperalgesia status of rats. Analgesic effect of moxibustion was similar to intrathecal injection of Ro 25-6981. The expression of NR1, NR2B, and PKC*ε* in the spinal dorsal horns of IBS visceral hyperalgesia rats increased. Moxibustion on the Tianshu and Shangjuxu acupoints might inhibit the visceral hypersensitivity, simultaneously decreasing the expression of NR1, NR2B, and PKC*ε* in spinal cord of IBS visceral hyperalgesia rats. Based on the above experimental results, we hypothesized NR1, NR2B, and PKC*ε* of spinal cord could play an important role in moxibustion inhibiting the process of central sensitization and visceral hyperalgesia state.

## 1. Introduction

Visceral pain is one of the major symptoms in functional gastrointestinal disease patients. It usually causes inconvenience and massive economic burden to this group of patients. Visceral pain is the most common in irritable bowel syndrome (IBS) patients [1]; therefore, relief or elimination of the patient's pain becomes the main purpose of treatment. Active exploration and popularization of effective measures for treating visceral pain in IBS are important goals in the medical field. With visceral hypersensitivity (visceral pain), nonnociceptive stimulation and nociceptive stimulation both induce peripheral and central level changes. Central sensitization is a current research hotspot and is considered a key factor in the development and progression of visceral hypersensitivity and its visceral hyperalgesia [2]. Therefore, studying the mechanism underlying the treatment of IBS visceral hypersensitivity by moxibustion through the perspective of central sensitization will be important in elucidating the mechanism underlying the development and progression of conduction and transduction of analgesic signals by moxibustion.

Currently, the treatment of IBS visceral pain by western medicine is mainly limited to symptomatic treatment; there are too many drugs and severe side effects [3]. Treatment of IBS visceral pain by moxibustion methods has a long history and rich clinical experience. In the clinic, moxibustion can effectively treat bowel disease with visceral pain and diarrhea symptoms [4–6]. There are many records of “abdominal pain” (similar to IBS visceral pain) in ancient medical books. The Tianshu and Shangjuxu acupoints are the two commonly and frequently used acupoints for treatment of IBS [7–9]. The combinatorial use of these two acupoints belongs to the classic He-Mu combination acupoints for treating bowel diseases in the clinic. This combination acupoint method matches the Lower He-Sea point of six organs and the Front MU acupoint. However, modern science has few studies on the pathway underlying the transduction of the stimulation signal of moxibustion on the treatment of IBS visceral pain. Therefore, it is necessary to study the mechanism underlying the treatment of IBS visceral pain by moxibustion on these bases to determine the scientific basis of the analgesic effect of moxibustion. Such research will provide laboratory data and a theoretic basis for the popularization of moxibustion therapy in the treatment of IBS visceral pain.

The N-methyl-D-aspartate (NMDA) receptor is a heteromeric complex that includes three different subunits, NR1, NR2, and NR3. The NR2B subunit can form a functional NMDA receptor only when interacting with NR1. The NR2B subunit plays an important role in the transmission of nociceptive signals at the spinal cord level and in the production and maintenance of hyperalgesia [10–12]. The NMDA receptor channel composed of NR1/NR2B has high Ca^2+^-selective permeability and temperature sensitivity. Tyrosine phosphorylation of NR2B can enhance the activity of the NMDA receptor channel and promote intracellular signal transduction. When the persistent nociceptive stimulation signals are transmitted to the spinal dorsal horn, the release of excitatory amino acids (mainly glutamate) will increase. Glutamate can bind to the NMDA receptor and activate ion channels to cause Ca^2+^ influx; after interaction with the protein kinase C (PKC) regulatory region, the catalytic region is turned on to activate PKC [13]. Activated PKC can phosphorylate various receptor proteins and ion channels (including the NMDA receptor). On the one hand, activated PKC relieves the blocking effect of Mg^2+^on NMDA receptor ion channels to open NMDA receptor ion channels at a relatively low potential condition to promote Ca^2+^ influx. On the other hand, it promotes the increase of NOS expression and NO production. Through the complex function of NO, the excitation of spinal neurons is changed and maintained at an excitatory status for a long time thus causing central sensitization [14]. Using this positive feedback amplification effect, neurons further transmit nociceptive stimulation signals to central nervous system [15] to induce hyperalgesia. As a subtype of PKC, PKC*ε* plays a certain regulatory role in the process of transduction of pain signals at the spinal cord level [16, 17]. The role of PKC in sensory afferents, especially the process of central pain transduction, has received much attention.

Therefore, blocking the spinal NR1, NR2B, and PKC*ε* signals may become an important pathway to prevent or inhibit the central sensitization process of the development of visceral pain. Here, we observed the changes in spinal NR1, NR2B, and PKC*ε* in IBS visceral hyperalgesia rats, and we compared the regulatory effects on spinal NR1, NR2B, and PKC*ε* in IBS visceral hyperalgesia rats treated with moxibustion versus intrathecal injection of Ro 25-6981, a selective antagonist of the NMDA receptor channel composed of NR1/NR2B. This study will improve our understanding of the signal conduction and transduction mechanisms underlying the regulation of central sensitization by moxibustion, which can help us investigate the analgesic mechanism of moxibustion.

## 2. Experimental Animals and Methods

### 2.1. Model Establishment

A total of 68 newborn male specific pathogen-free- (SPF-) grade SD rats (5 days old) were provided by the Experimental Animal Center of Shanghai University of Traditional Chinese Medicine. Lactating rats were given free access to food and water. The housing environment was 12 h light/12 h dark, 20 ± 2°C, and 50–70% humidity. After adaptive feeding for 3 days, the experiments began. During the experimental process, animal treatment conformed to the guidelines of the International Association for the Study of Pain (IASP).

Using the complete randomization method, the 68 8-day-old neonatal rats were divided into the normal control group (*n* = 18) and the model group (*n* = 50). Neonatal rats in the normal group only received genital scratching without other stimuli. For neonatal rats in the model group, colorectal stimulation balloons for IBS visceral hyperalgesia were prepared according to the method of Al-Chaer et al. [18]. Colorectal distension (CRD) was performed in awake animals. During the operation, liquid paraffin was applied to the surface of each balloon. The balloon was gently inserted anally along the physiological curvature of rectum to the descending colon at a depth of approximately 2 cm. At this point, the balloon began to inflate to 0.2 mL over 1 min. The injector was slowly withdrawn, and the balloon was removed. After 1 hour, the same stimulation was repeated once. The balloon stimulation was performed once every day at a fixed time for 14 days. The balloon stimulation was not performed for the next 21 days. Rats were continuously fed until week 6, when the experimental grouping and processing were performed.

The AWR scoring standards followed the method of Li et al. [19] (Table 1). CRD stimulation used homemade balloons that were connected to a desktop sphygmomanometer and an injector through a three-way valve. Before scoring, animals were fasted for food but not water for 8–12 h to reduce the formation of feces. Before distension stimulation, the head end of the balloon gently touched the anus to facilitate defecation by rats. The balloon was gently inserted anally along the physiological curvature of rectum to the descending colon. The injector was pushed to provide constant pressure for distension stimulation. The sphygmomanometer connected to the three-way valve was used to control the pressure of balloon dilation. Four different pressures, 20 mmHg, 40 mmHg, 60 mmHg, and 80 mmHg, were supplied to perform CRD stimulation. Three AWR scores were recorded for each rat. CRD stimulation was performed for 20 s each time at 5-minute intervals. The mean of the three AWR scores was used as the final score. Within 60 min of the last moxibustion treatment, CRD was performed to observe the AWR scores of rats in each group (Figure 2).

### 2.2. Animal Grouping

**Figure 1:**
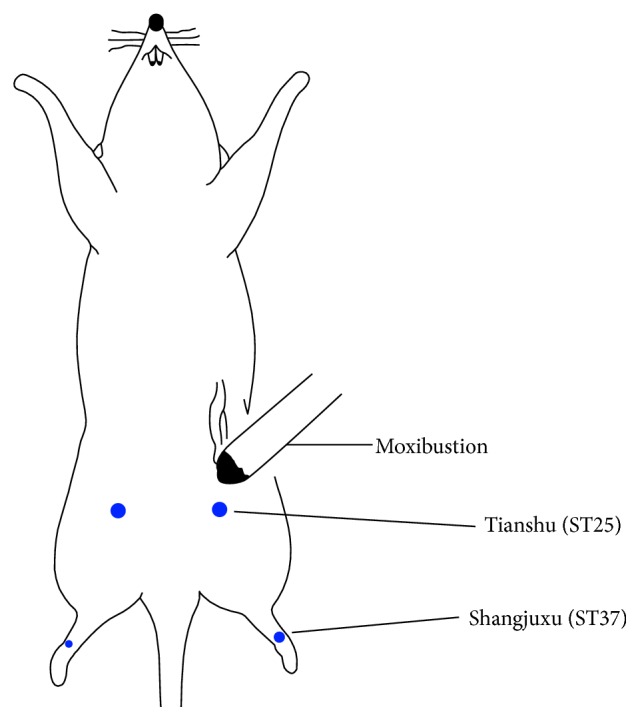
Diagram of moxibustion in rats. Rats were immobilized on a fixing frame. A moxa stick was lighted, and the lighted end was placed perpendicularly to the acupoint, approximately 2 cm above it. This was performed for 10 min once every day for 7 days.

After the model was successfully established, rats in the model group were grouped using the complete randomization method into the model group, moxibustion group, and Ro 25-6981 group. (1) The normal group (*n* = 16) rats were normally treated without CRD stimulation. (2) The model group (*n* = 16) rats were not treated after the model was established successfully. (3) The moxibustion group (*n* = 16) received stimulation at the “Tianshu” (bilateral) and “Shangjuxu” (bilateral) acupoints (the position of the acupoints followed Experimental Acupuncture [20] and relevant literature [21, 22]). Rats were immobilized on a fixing frame. A moxa stick was lighted, and the lighted end was placed perpendicularly to the acupoint, approximately 2 cm above it. This was performed for 10 min once every day for 7 days [23] (Figure 1). The Tianshu point was approximately 5 mm away from the Shenque acupoint (the distance to subxiphoid area and pubic symphysis at a ratio of 8 : 5, i.e., 8 from the top and 5 from the bottom). The Shangjuxu acupoint was 1 mm from the line that connected the horizontal surface of the medial tibial condyle and external malleolus at 6 : 10 (6 from the top and 10 from the bottom). (4) The Ro 25-6981 group (*n* = 16) received intrathecal injection of 5 *µ*g/*µ*L Ro 25-6981 [11, 24–26]. The treatment was performed once every day for 7 days. Rats were anesthetized by intraperitoneal injection of 2% pentobarbital sodium at 0.25 mL/100 g. The fur at the waist was shaved, and disinfection was performed using 75% alcohol cotton balls. Rats were placed in the prone position. The left thumb and middle finger were placed at both sides of the interval between L5 and L6 of the spinal cord. The right hand held a microinjector to aspirate 20 *µ*L Ro 25-6981. The needle was slowly inserted perpendicularly to the spinous process interval. The quiver or lateral swing of the rat tail was a marker of successful puncture. The drug was slowly injected.

### 2.3. Specimen Collection and Processing

After the treatment was finished, intraperitoneal injection of 2% pentobarbital sodium at 0.25 mg/100 g was performed immediately to anesthetize animals and collect specimens. Specimens were prefixed in 4% paraformaldehyde, and the descending colon at 10 cm above the anus was collected. The colon was cut at 3 cm from its end, the intestinal tube was cut open longitudinally, and other debris such as the mesenteric membrane and connective tissues were removed. Feces on the intestinal wall were washed using PBS, and specimens were fixed in preprepared 4% paraformaldehyde. The L6-S2 segment of the spinal cord was used for western blot and real-time quantitative polymerase chain reaction (PCR).

### 2.4. Real-Time PCR

Total RNA was extracted from the L6-S2 segment of spinal dorsal horn tissues. cDNA was synthesized using the reverse transcriptase reaction system (Jrdun Biotechnology (Shanghai) Co., Ltd.). The relative gene expression level was controlled using the ΔΔCT method. Data were analyzed using the ABI Prism 7300 SDS software.

### 2.5. Immunohistochemistry

NR1, NR2B, and PKC*ε* in rat spinal cord were detected using immunohistochemistry. Antigen retrieval was performed by heating samples in 0.01 M citric acid buffer (pH 6.0) in a microwave oven. After washing with 0.01 M PBS (pH 7.2–7.6) for 3 × 5 min, specimens were treated with 3% H_2_O_2_ in the dark for 15 min to inactivate endogenous peroxidases. After washing with 0.01 M PBS for 3 × 5 min, specimens were immersed in the diluted anti-NMDAR1 antibody, anti-PKC epsilon antibody, or anti-NMDAR2B antibody (Abcam, UK) and incubated at 4°C overnight. The specimens were warmed up for 30 min, washed with 0.01 M PBS for 3 × 5 min, and incubated with the secondary antibody from the EnVision reagent kit (Gene Tech (Shanghai) Co., Ltd., Shanghai, China) at room temperature for 30 min. After washing with 0.01 M PBS for 3 × 5 min, DAB developing solution (Gene Tech (Shanghai) Co., Ltd., Shanghai, China) was added, the tissues were washed with double-distilled water to terminate the development, and then they were dehydrated, mounted, and observed under a light microscope.

### 2.6. Western Blot

NR1, NR2B, and PKC*ε* were measured by western blot in spinal cord tissues of rats in all groups. RIPA lysis buffer at the ratio of 100 mg tissue/mL lysis buffer (Beyotime Institute of Biotechnology, China) was used for the extraction of total protein from spinal dorsal horns. The protein concentration was determined using the BCA method with the BCA protein concentration determination reagent kit (Beyotime Institute of Biotechnology, China). Protein standards at 0.5 mg/mL were added to standard wells in a 96-well plate, and the volume was filled up to 40 *μ*L using the standard dilution buffer. The absorbance value at 562 nm of each well was measured. According to the standard curve, the protein concentrations of samples were calculated. The loading mass of samples was 100 *μ*g and the loading volume was 10 *μ*L. Samples were subjected to sodium dodecyl sulfate-polyacrylamide gel electrophoresis (SDS-PAGE) (Bio-Rad, USA) and transferred to a nitrocellulose (NC) membrane (wet transfer). After the transfer was finished, the NC membrane was removed, placed in an incubation box, and blocked in an appropriate amount of 5% BSA on a shaker at room temperature for 1 h. The trimmed NC membrane was placed in a corresponding incubation box and incubated with PKC*ε* rabbit mAb, NMDAR1 rabbit mAb, NMDAR2B rabbit mAb (Cell Signaling Technology, USA), and HRP-labeled *β*-actin internal control followed by HRP-labeled goat anti-rabbit IgG secondary antibody (Beyotime Institute of Biotechnology, China) (according to the titers of antibodies, they were diluted in 5% BSA at 1 : 1000). The A and B solutions (500 *μ*L each) from the ECL chemiluminescence reagent kit (Pierce) were mixed in a centrifuge tube. The NC membrane incubated with HRP-labeled *β*-actin was dipped into the A and B solution mix for 1 min. The chemiluminescence solution was removed, and the membrane was automatically exposed in a gel documentation system. The software in the system was used to analyze the target protein bands.

### 2.7. Statistical Methods

Experimental data were all analyzed using SPSS 19.0. If experimental data conformed to the normal distribution and had homogeneous variances, the statistical description was presented as $\overset{-}{x}\pm s$. The comparison of mean values between two independent samples was statistically analyzed using the two-sample *t*-test. The comparison of mean values between multiple independent samples was examined using the one-way analysis of variance (ANOVA). *P* < 0.05 implied statistical significance.

## 3. Experimental Results

### 3.1. AWR Scores of Rats in Each Group after Moxibustion Intervention

**Figure 2:**
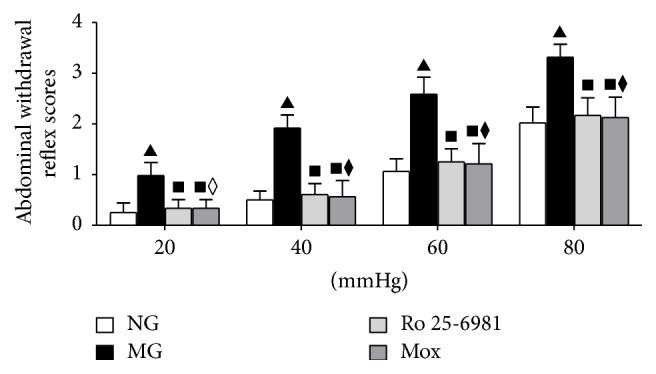
AWR scores of rats in each group after moxibustion intervention. Under the same intensity of CRD stimulation, compared to the normal group, ^▲^ *P* < 0.001; compared to the model group, ^■^ *P* < 0.001; compared to the Ro 25-6981 group, ^⧫^ *P* < 0.05, ^◊^ *P* > 0.05.

After moxibustion, under different pressure levels of CRD stimulation, the AWR scores were significantly different in all intergroup comparisons (*P* < 0.001). Multiple comparisons using the Nemenyi method showed that, with the gradual increase of the stimulation intensity, the AWR scores of rats in each group increased accordingly. Under the same intensity of pressure stimulation, the AWR score in the model group significantly increased compared to the normal group (*P* < 0.001). Compared to the model group, the AWR scores in the moxibustion group and the Ro 25-6981 group both significantly decreased (*P* < 0.001). Figure 2 shows that moxibustion significantly decreased the AWR score of IBS visceral hyperalgesia rats and effectively relieved the IBS visceral hyperalgesia status of rats.

### 3.2. Pathology of Colon Tissues (HE Stain)

**Figure 3:**
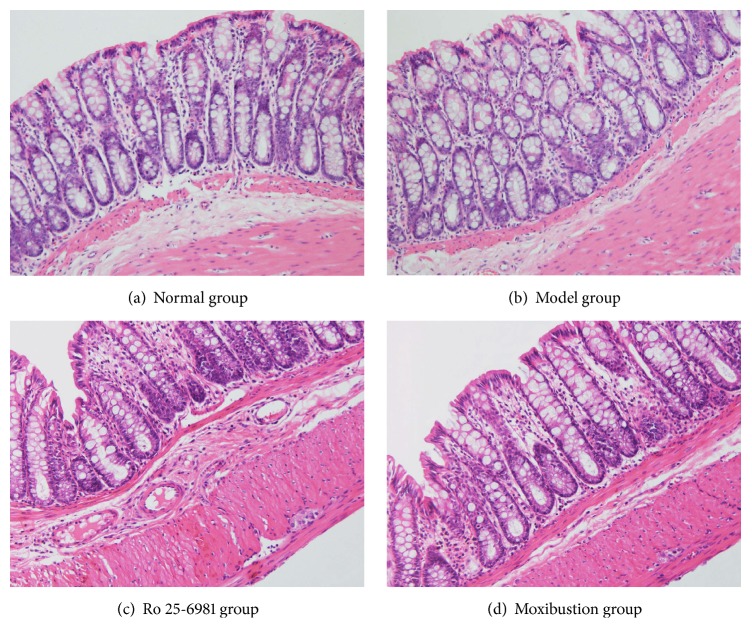
Pathology of colon tissues (HE stain ×200). The observation of colon tissues under a light microscope showed that the overall structure of colon tissues of rats in the model group was clear. There were no abnormal pathological changes such as hyperplasia, erosions, or ulcers. There was no significant inflammatory cell infiltration and no obvious interstitial edema. The mucosal epithelium was complete, the glands in the lamina propria were arranged orderly, and the distribution of submucosa layer and muscular layer was regular.

The observation of colon tissues under a light microscope showed that the overall structure of colon tissues of rats in the model group was clear (Figure 3). There were no abnormal pathological changes such as hyperplasia, erosions, or ulcers. There was no significant inflammatory cell infiltration and no obvious interstitial edema. The mucosal epithelium was complete, the glands in the lamina propria were arranged orderly, and the distribution of submucosa layer and muscular layer was regular. These results conformed to the clinical characteristics of IBS that there was abdominal pain but there were no obvious histopathological changes.

### 3.3. Expression of NR1, NR2B, and PKC*ε* mRNA in Spinal Cord

The above experiments showed that the NR1, NR2B, and PKC*ε* receptor proteins played important roles in the pathogenesis of visceral pain and the relief of visceral pain by moxibustion. In particular, intrathecal injection of Ro 25-6981 inhibited the expression of the NR1 and NR2B subtypes; at the same time, the visceral hyperalgesia was also significantly decreased. To observe whether the mRNAs of the NR1, NR2B, and PKC*ε* subtypes also changed during this process, real-time PCR was used.

**Figure 4:**
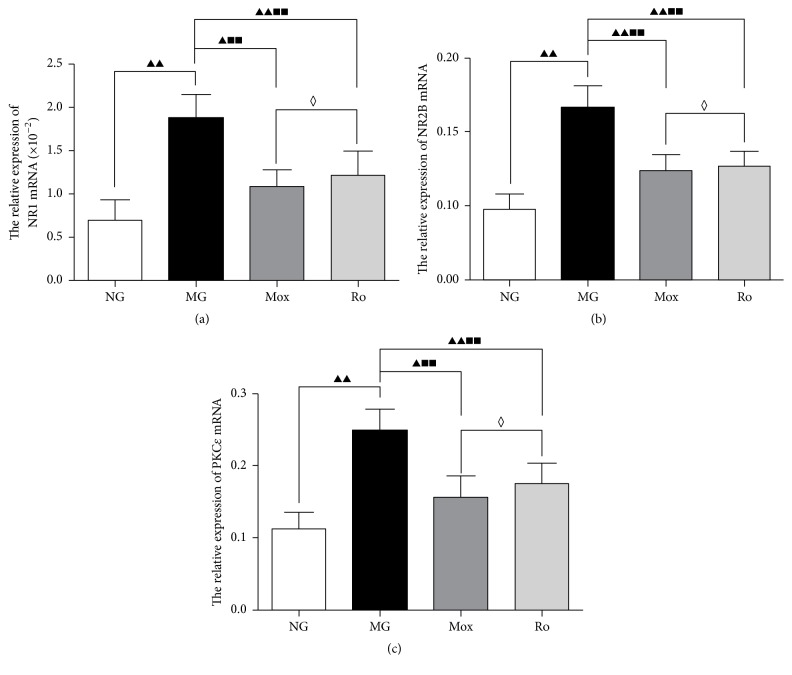
NR1 mRNA expression in the spinal cord was significantly different between all groups (*P* < 0.001) except the moxibustion versus the Ro 25-6981 group (*P* > 0.05) (a). NR2B (b) and PKC*ε* (c) followed the same pattern. Pairwise comparisons by LSD *t*-test. Compared to the normal group, ^▲▲^ *P* < 0.001 and ^▲^ *P* < 0.01; compared to the model group, ^■■^ *P* < 0.001; compared to the Ro 25-6981 group, ^◊^ *P* > 0.05.

Real-time PCR showed that NR1 and NR2B were upregulated in spinal cord tissues of rats in the model group compared to the normal group (*P* < 0.001) (Figures 4(a) and 4(b)); the level of upregulation of NR2B mRNA was higher than that of NR1 mRNA. Compared to the model group, moxibustion (Tianshu and Shangjuxu) and intrathecal injection of Ro 25-6981 both inhibited the expression of NR1 and NR2B mRNA in the spinal cord of rats (*P* < 0.001). The comparison between the moxibustion group and the Ro 25-6981 group did not show a significant difference (*P* > 0.05).

Based on the inhibitory effects of moxibustion and the NMDA receptor antagonist Ro 25-6981 on NR1 and NR2B mRNA and protein in the spinal dorsal horn, we observed whether moxibustion also had regulatory effects on PKC*ε* mRNA in the central spinal dorsal horns. The effects of moxibustion and Ro 25-6981 on PKC*ε* mRNA in the spinal cord tissues in rats were observed using real-time PCR. Compared to the normal group, PKC*ε* mRNA expression significantly increased in the model group (*P* < 0.001). Compared to the model group, PKC*ε* mRNA expression in the moxibustion group and the Ro 25-6981 group significantly decreased (*P* < 0.001) (Figure 4(c)), suggesting that PKC*ε* mRNA expression significantly increased in the spinal cord of IBS visceral hyperalgesia rats and that moxibustion mitigates this increase.

### 3.4. Immunohistochemistry for NR1, NR2B, and PKC*ε* in Spinal Dorsal Horn

**Figure 5:**
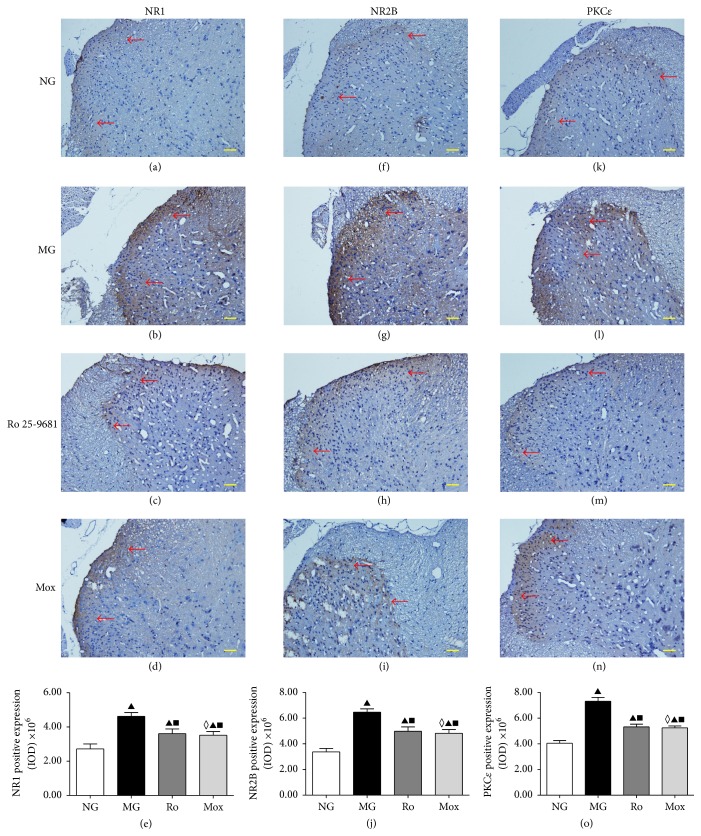
The differences in the expression of NR1 ((a) and (d)), NR2B ((f)–(i)), and PKC*ε* ((k)–(n)) in spinal cord were statistically significant between all groups (*P* < 0.001) ((e), (j), and (o)). The least significant difference (LSD) *t*-test was performed for pairwise comparison, and the results showed that the expression of NR1, NR2B, and PKC*ε* in the model group significantly increased compared to the normal group (*P* < 0.001) ((b), (g), and (l)). Compared to the model group, the expression of NR1, NR2B, and PKC*ε* in the moxibustion group ((d), (i), and (n)) and the Ro 25-6981 group ((c), (h), and (m)) significantly decreased (*P* < 0.001) ((e), (j), and (o)). Compared to the normal group, ^▲^ *P* < 0.001; compared to the model group, ^■^ *P* < 0.001. The comparison between the moxibustion group and the Ro 25-6981 group did not show a significant difference (^◊^ *P* > 0.05) (magnification ×200).

The immunohistochemistry was performed in spinal tissues with positive expression of NR1, NR2B, and PKC*ε* to detect their localization and for semiquantitative detection. Each pairwise comparison between groups showed significantly different levels of positive immunoactive expression of NR1 subtypes in the cell membrane and cytoplasm of neurons in the spinal dorsal horn (*P* < 0.001) (Figures 5(a)–5(d)). As shown in Figure 5(e), the expression was concentrated in layers I and II of the spinal dorsal horn. The positive expression of the NR1 subtype was yellow to brown, and the cell nucleus was blue. In the normal group, NR1 had weak expression in layers I and II; the color was light and the distribution was sparse (Figure 5(a)). In the model group, the NR1-positive staining was darker than that in the normal group (*P* < 0.001) and the distribution was denser (Figure 5(b)). In the Ro 25-6981 and moxibustion groups, the NR1-positive expression was weaker than that in the model group (*P* < 0.001); the staining was lighter and the distribution was sparser (Figures 5(c) and 5(d)). The comparison between the moxibustion group and the Ro 25-6981 group did not show a significant difference (*P* > 0.05).

Positive immunoactive expression of NR2B in the cytoplasm and cell membrane of spinal dorsal horn neurons was also different in each group (*P* < 0.001) (Figures 5(f) and 5(i)). NR2B stained yellowish brown. Figure 5(j) shows that its location was similar to that of the NR1 subtype: it was concentrated in layers I and II of the dorsal horn. In the normal group, NR2B had weak expression in layers I and II; the color was light and the distribution was sparse (Figure 5(f)). The NR2B-positive staining in the model group was darker than that in the normal group (*P* < 0.001) and the distribution was denser (Figure 5(g)). The NR2B-positive expression in spinal cord of rats in the Ro 25-6981 group and the moxibustion group significantly decreased compared to the model group (*P* < 0.001); the color was lighter and the distribution was sparser (Figures 5(h) and 5(i)).

These results suggest that NR1 expression in spinal cord in IBS visceral hyperalgesia rats significantly increases and that moxibustion could significantly reduce the expression of NR1 and NR2B in IBS visceral hyperalgesia rats.

Immunohistochemistry for PKC*ε* in the spinal dorsal horn neurons showed different levels of immunoactive expression in the cell membrane and cytoplasm between all four groups (*P* < 0.001) (Figures 5(k) and 5(n)). As shown in Figure 5(o), the expression was again concentrated in layers I and II. The expression of the PKC*ε* subtype was yellow to yellowish brown, and the nucleus was blue. The PKC*ε*-positive staining in the model group was darker than that in the normal group (*P* < 0.001) and the distribution was denser (Figures 5(k) and 5(l)). The PKC*ε*-positive staining in the Ro 25-6981 and moxibustion groups decreased compared to the model group (*P* < 0.001); the color was lighter and the distribution was sparser (Figures 5(m) and 5(n)). The comparison between the moxibustion group and the Ro 25-6981 group did not show a significant difference (*P* > 0.05). The expression change of the PKC*ε* subtype had a similar trend to that of the NR1 and NR2B subtypes, suggesting that PKC*ε* expression in the spinal cord of IBS visceral hyperalgesia rats significantly increases and that moxibustion mitigates this increase.

### 3.5. Western Blot for NR1, NR2B, and PKC*ε* in Spinal Dorsal Horn

Based on the above localization and semiquantitative detection, western blot was performed to quantify NR1, NR2B, and PKC*ε* in the spinal dorsal horn in all four groups.

**Figure 6:**
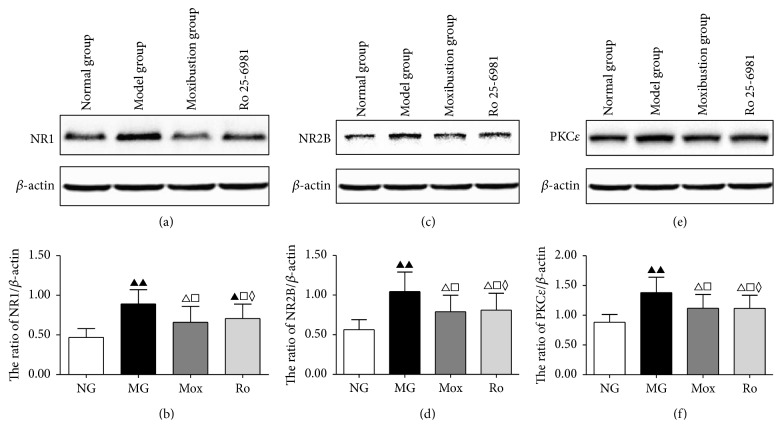
The levels of NR1 and NR2B proteins in spinal cord of rats were different between all groups (*P* < 0.001) ((a), (b), (c), and (d)). Further pairwise comparison using the LSD *t*-test showed that the expression of NR1 and NR2B proteins in the model groups significantly increased compared to the normal group (*P* < 0.001). Compared to the model group, NR1 and NR2B proteins in the moxibustion group and Ro 25-6981 group both significantly decreased (*P* < 0.05). PKC*ε* showed a similar pattern. ((e) and (f)) compared to the normal group, ^▲▲^ *P* < 0.001, ^▲^ *P* < 0.01, and ^△^ *P* < 0.05; compared to the model group, ^□^ *P* < 0.05; compared to the Ro 25-6981 group, ^◊^ *P* > 0.05.

Detection of NR1 and NR2B in the spinal cord using western blot showed increased expression in the model group compared to the normal group (*P* < 0.001) (Figures 6(b) and 6(d)). Compared to the model group, moxibustion on the Tianshu and Shangjuxu acupoints and Ro 25-6981 treatment both downregulated NR1 and NR2B proteins in spinal cord (*P* < 0.05). The comparison between the moxibustion group and the Ro 25-6981 group did not show a significant difference (*P* > 0.05). These results suggest that the expression of NR1 and NR2B protein significantly increases in the spinal cord of IBS visceral hyperalgesia rats and that moxibustion on the Tianshu and Shangjuxu acupoints reverses this increase.

Detection of PKC*ε* in the spinal cord showed a significant increase in the model group compared to the normal group (*P* < 0.001) (Figure 6(f)). Compared to the model group, moxibustion on the Tianshu and Shangjuxu acupoints and Ro 25-6981 both downregulated PKC*ε* in spinal cord (*P* < 0.05). The comparison between the moxibustion group and the Ro 25-6981 group did not show a significant difference (*P* > 0.05). These results suggest that the expression of PKC*ε* protein significantly increases in the spinal cord of IBS visceral hyperalgesia rats and that moxibustion on the Tianshu and Shangjuxu acupoints reversed this increase.

## 4. Discussion

Central sensitization is considered a key factor in the process of development and progression of visceral hypersensitivity and visceral hyperalgesia. The spinal dorsal horn is the first relay station of the transmission of pain signals into the central nervous system. It can both directly regulate pain messages and receive the downward regulatory signals from the central nervous system above the spinal cord to regulate the transmission of nociceptive signals received by internal organs. Therefore, the spinal dorsal horn has received extensive attention in the studies of central sensitization. Moxibustion on the Tianshu acupoint can significantly reduce the AWR score of IBS rats and improve the abnormally high PK1 and PKR1 in spinal cord and colon tissues [27, 28]. Moxibustion treatment can increase the levels of endogenous opioid peptides (dynorphin, endomorphin, enkephalins, and orphanin), suggesting that these peptides at the spinal cord level might be involved in the analgesic effect of moxibustion [29, 30]. The analgesic mechanism of moxibustion is more complex, involving the nervous-endocrine-immune aspects and being associated with parameters such as acupoint specificity, stimulation time, and temperature.

Boyce et al. [31] showed that the distribution of NR2B is mainly limited to the spinal dorsal horns, especially layers I and II. Nagy et al. [32] used the pepsin digestion technique to expose antigens and observed the distribution of NR2B in layers I and II during visceral pain. NR1 is distributed throughout the central nervous system. The expression level of NR1 significantly increases in lamina layers I and II of the spinal cord dorsal horn in rats with visceral hypersensitivity induced by intracolonic trinitrobenzene sulfonic acid (TNBS) [33]. The NR2B subunit plays an important role in the transmission of nociceptive messages at the spinal cord level and the generation and maintenance of hyperalgesia [34]. In the thoracolumbar segment and lumbosacral segment of rats in the IBS model, the expression of NR2B protein significantly increases [35], indicating that the chronic visceral hyperalgesia in IBS-like rats might be associated with the increased NR2B subunit in these two segments. These results suggest that NR2B might play an important role in the process of transmission of chronic visceral pain signals.

We used the CRD stimulation method to prepare a rat model of IBS visceral hyperalgesia. The behavioral tests showed that, under different pressure levels of CRD stimulation, the AWR scores in the model groups all significantly increased, indicating visceral hypersensitivity. HE staining results also suggested that the colon in the model group did not have obvious pathological changes, which confirmed the clinical characteristics of IBS of abdominal pain but no significant histopathological changes. The expression of NR1 protein and mRNA in layers I and II of the spinal dorsal horn was higher than in normal rats (Figures 4(a), 5(b), and 6(b)). The analysis of NR2B protein and mRNA produced similar results (Figures 4(b), 5(g), and 6(d)). However, the expression levels of NR2B protein and mRNA were significantly higher than those of NR1. Combined with the observation of the behavioral scores of rats (Figure 2), these results indicate that NR1 and NR2B might play a role in the pathogenesis of the increase of visceral sensitivity.

The NMDA receptor in the central nervous system is involved in the formation of visceral hyperalgesia-central sensitization [36, 37]. Intrathecal injection of NMDA enhances the pain reaction of animals induced by internal organ stimulation in a dose-dependent manner. Electrophysiological experiments also suggest that NMDA induces the enhancement of discharge by neurons upon CRD stimulation, which is also combined with a strong afterdischarge [38]. Intrathecal injection of specific NMDA receptor antagonists could relieve visceral hypersensitivity in rats [39]. To further validate this result, we performed intrathecal injection of the NMDA receptor antagonist Ro 25-6981 to block the NMDA receptor function above the spinal cord level. First, the AWR score of the visceral reflex of IBS rats was measured. Intrathecal injection of Ro 25-6981 significantly decreases the visceral hyperalgesia (Figure 2). Detection of NR1 and NR2B in the spinal dorsal horns of rats in the Ro 25-6981 group also showed decreased NR1 and NR2B mRNA (Figures 4(a), 4(b), 5(e), 5(j), 6(b), and 6(c)). After moxibustion treatment, the visceral hyperalgesia of rats in the model group was significantly relieved (Figure 2). We speculated that moxibustion exerted its analgesic effect through the regulation of NR1 and NR2B function. We therefore detected the mRNA expression of NR1 and NR2B in the spinal dorsal horn and found that moxibustion mitigated the increases in NR1 and NR2B mRNA seen in the model rats (Figures 4(a), 4(b), 5(e), 5(j), 6(b), and 6(d)). These results indicate that, by inhibiting NR1 and NR2B expression in layers I and II of the dorsal horn, moxibustion inhibits the binding between excitatory glutamate and NMDA receptor and decreases the number of activated ion channels, thus inhibiting the function of the regulatory region of PKC and eventually relieving visceral pain. In this study, the efficacy of moxibustion on the relief of visceral pain was slightly better than that of the intrathecal injection of Ro 25-6981. One possible reason was that moxibustion also relieved visceral pain through other pathways.

Activated PKC can phosphorylate many receptors and ion channels, including NMDA receptor. As a subtype of PKC, PKC*ε* plays a certain regulatory role in the process of transduction of pain signals at the spinal cord level. Sweitzer et al. [40] showed that PKC*ε* might regulate the phenomenon of hypersensitive response or excessive pain reaction to stimulation presented by patients after morphine withdrawal in clinical setting. Zhang et al. [41] used western blot to study the function of PKC*ε* in visceral pain through a rat model of visceral pain. They found that PKC*ε* is activated during the process of visceral pain, suggesting that PKC*ε* might be involved in the development of visceral pain. In the present study, layers I and II of the spinal dorsal horn of IBS rats induced by mechanical CRD stimulation had upregulated PKC*ε* protein and mRNA (Figures 4(c), 5(o), and 6(f)). Our previous experiments had already shown that moxibustion could regulate the stage of binding between excitatory glutamate and the NR1 and NR2B subtypes of the NMDA receptor; the intrathecal injection of the NMDA receptor antagonist downregulated PKC*ε* in layers I and II of the spinal dorsal horn (Figure 5(o)). These results indicate that the function of PKC*ε* is affected after the NMDA receptor is inhibited. We speculated that moxibustion also regulates PKC*ε* activation to exert its effects on relieving visceral pain. In the group treated by moxibustion on the Tianshu and Shangjuxu acupoints seven times, PKC*ε* protein and mRNA were downregulated in layers I and II of the spinal cord dorsal horn (Figures 4(c), 5(o), and 6(f)), indicating that PKC*ε* is involved in the relief of visceral pain by moxibustion.

In summary, by decreasing the expression of NR1, NR2B, and PKC*ε* in spinal cord of IBS visceral hyperalgesia rats, moxibustion reduced the transduction of visceral hyperalgesia signals and inhibited the process of central sensitization, which might be an important mechanism by which moxibustion relieves the IBS visceral hyperalgesia status.

## Figures and Tables

**Table 1:** Abdominal withdrawal reflex (AWR) scoring criteria.

Score 0	No behavioral response to colorectal distension (CRD)

Score 1	Immobility during distension of colorectum (CR) and occasional appearance of brief head motion after a pause at the onset of the stimulation

Score 2	A mild contraction of abdominal muscles, but no lifting of the abdomen off the platform

Score 3	A strong contraction of abdominal muscles and lifting of abdomen off the platform, no lifting of pelvic structure off the platform

Score 4	Arching body and lifting of pelvic structure and scrotum
